# Osteoimmunology: Major and Costimulatory Pathway Expression Associated with Chronic Inflammatory Induced Bone Loss

**DOI:** 10.1155/2015/281287

**Published:** 2015-05-03

**Authors:** Tania N. Crotti, Anak A. S. S. K. Dharmapatni, Ekram Alias, David R. Haynes

**Affiliations:** ^1^Discipline of Anatomy and Pathology, School of Medical Sciences, The University of Adelaide, Adelaide, SA 5005, Australia; ^2^Department of Biochemistry, Faculty of Medicine, Universiti Kebangsaan Malaysia, Jalan Yaacob Latif, Bandar Tun Razak, 56000 Kuala Lumpur, Malaysia

## Abstract

The field of osteoimmunology has emerged in response to the range of evidences demonstrating the close interrelationship between the immune system and bone metabolism. This is pertinent to immune-mediated diseases, such as rheumatoid arthritis and periodontal disease, where there are chronic inflammation and local bone erosion. Periprosthetic osteolysis is another example of chronic inflammation with associated osteolysis. This may also involve immune mediation when occurring in a patient with rheumatoid arthritis (RA). Similarities in the regulation and mechanisms of bone loss are likely to be related to the inflammatory cytokines expressed in these diseases. This review highlights the role of immune-related factors influencing bone loss particularly in diseases of chronic inflammation where there is associated localized bone loss. The importance of the balance of the RANKL-RANK-OPG axis is discussed as well as the more recently appreciated role that receptors and adaptor proteins involved in the immunoreceptor tyrosine-based activation motif (ITAM) signaling pathway play. Although animal models are briefly discussed, the focus of this review is on the expression of ITAM associated molecules in relation to inflammation induced localized bone loss in RA, chronic periodontitis, and periprosthetic osteolysis, with an emphasis on the soluble and membrane bound factor osteoclast-associated receptor (OSCAR).

## 1. Introduction

The term osteoimmunology and the study of osteoimmunology have developed due to the close interrelationship between the immune system and bone metabolism [1]. This is evident in immune-mediated diseases, such as rheumatoid arthritis and periodontal disease (periodontitis), where there are local bone erosion and inflammation as reviewed in detail in multiple publications [2–4]. Similarities in the mechanisms of bone loss in disease are likely related to the inflammatory cytokines expressed in a number of bone loss diseases. These cytokines are known to upregulate osteoclast activity via increased expression levels of receptor activator NF kappa B ligand (RANKL) relative to osteoprotegerin (OPG) (as explored below) and increase localized bone loss in diseases such as RA, periodontal disease, and periprosthetic osteolysis [5–10].

This review highlights the role of immune-related cells and factors in modulating bone loss, particularly in these diseases. While the importance of the RANKL-RANK-OPG axis has been appreciated for nearly two decades [11–13], more recent studies have highlighted the importance of factors associated with immunoreceptor tyrosine-based activation motif (ITAM) signalling. This review will briefly discuss the RANKL-RANK-OPG axis but its major focus will be on the role of ITAM-associated factors, the more recently investigated pathway, and how it relates to inflammatory bone loss diseases, in particular osteoclast-associated receptor (OSCAR) [14].

## 2. Chronic Inflammation-Mediated Bone Loss

### 2.1. Rheumatoid Arthritis

Rheumatoid arthritis (RA) affects 1-2% of the population and involves an autoimmune reaction with an autoantibody response to citrullinated proteins (and others such as rheumatoid factor and collagen type II) [15]. RA is characterized by synovitis involving angiogenesis, synovial proliferation, increased infiltration, survival, and decreased apoptosis of inflammatory cells [16]. Further to this there is an increase in osteoclast number and activity leading to focal bone erosions, juxta-articular osteopenia, and joint destruction [17–20]. Animal models suggest that there may also be suppression of localised osteoblast formation of bone [21].

### 2.2. Periodontitis and Similarities to RA

Periodontitis is a chronic inflammatory disease of the gingival tissues, with an associated loss of the supporting structures including the periodontal ligament and alveolar bone. The aetiology involves an inflammatory response to bacterial infection such as* P. gingivalis* and possibly an autoimmune reaction, as reviewed [22]. Periodontitis is the most common and widespread bone loss pathology in humans with 64% of the US population aged 65 years and older reported as having moderate or severe periodontitis [23]. Despite the prevalence of this disease the most common treatment is either mechanical subgingival plaque removal or surgical debridement. Inevitably, in the absence of effective treatment, support structures (periodontium) are compromised and the affected teeth will loosen and fall out.

RA and periodontitis have a similar pathophysiology, characterized by destructive inflammation that culminates in localized bone loss. The citrullination of proteins by* P. gingivalis* and the subsequent generation of autoantigens that drive autoimmunity in RA have been proposed as a possible mechanism linking these two diseases [24]. Similarities in RA and periodontitis may relate to citrullinated enolase as the specific antigen involved as well as cross-reaction between the antibodies directed towards the immunodominant epitope of human citrullinated alpha-enolase and a conserved sequence on citrullinated* P. gingivalis* enolase [25].

New evidence suggests a relationship between the extent and severity of chronic periodontitis and RA [24, 25]. Individuals with advanced RA are more likely to experience more significant periodontal problems compared to their non-RA counterparts and vice versa. This is supported by findings in a study using a combined animal model of RA and periodontitis [26], which demonstrated more severe development of arthritis in mice with periodontitis. Further to this, mice in which periodontitis alone was induced had evidence of radiocarpal bone loss in the absence of arthritic disease [26]. Additionally, mice in which inflammatory arthritis was induced also had evidence of periodontitis [26]. This suggests presence of either RA or periodontitis places the individual at risk of developing the other disease. Both conditions involve an imbalance between proinflammatory and anti-inflammatory cytokines and increased bone-resorbing activity. Cantley et al. (2011) thus proposed that these two diseases are related through a common underlying dysfunction of fundamental inflammatory mechanisms [26]. New treatment strategies are needed for both diseases that target the inhibition of proinflammatory cytokines, destructive enzymes, and bone-resorbing activity. The clinical implications of the current research strongly suggest that patients with RA should be carefully screened for their periodontal status, as reviewed [27].

### 2.3. Peri-Prosthetic Osteolysis

Joint replacement surgery is used as a last resort in osteoarthritis (OA) and RA patients and is a relatively successful operation; however, a large proportion of implants fail within 10–20 years as a result of bone loss and implant loosening [4, 10, 28]. The pathogenesis behind prosthetic implant failure involves wear of prosthetic alloys, such as polyethylene (PE), cobalt chrome, and titanium liberated from the implant surface [29–31]. These particles stimulate a chronic inflammatory response [29], which increases bone-resorbing activity of the osteoclasts [32] and suppresses bone formation by the osteoblast [33, 34] resulting in bone loss [25]. Periprosthetic tissues contain granulomatous lesions dominated by inflammatory cells, particularly macrophages, and foreign-body giant cells [29–31, 35, 36]. It is believed that an inflammatory reaction is initiated within the tissues in an attempt at particle clearance. This then becomes a chronic reaction resulting in a granulomatous lesion. This granulomatous lesion in periprosthetic osteolysis often leads to the formation of pseudosynovium-like structure, in which cells are organized into lining layer, in the membranous tissues adjacent to the failed implant surface [35]. Juxtaposed to this pseudosynovium are fibrous and collagenous regions, possibly scar tissues, which could be indicative of late stage periprosthetic osteolysis. The plethora of factors release in this inflammatory reaction within the tissues contributes towards the promotion of osteoclast formation [9, 37].

Higher numbers of T lymphocytes have been observed in the periprosthetic tissues of human and mouse models containing polyethylene and metal particles compared with normal tissues [38–40] and osteoarthritic tissues [41]. Sandhu et al. (1998) proposed that T cells are indirectly affected by the inflammatory cascade induced by wear particles [40]. It is however important to note that T cells make up less than 10% of total cell population [42] in periprosthetic tissues. Given the low levels of T lymphocytes, the general belief held is that lymphocyte infiltrates are not normally associated with wear-particle induced periprosthetic osteolysis, in particular, in the granulomatous region [29, 32, 42, 43]. The role of T cells may however be more pertinent in RA patients with implants. The prevalence of foreign-body giant cells in response to implant-derived wear debris in RA patients and non-RA patients does not differ but appears to be linked to the amount of polyethylene wear debris [36]. As would be expected, this study reported a high prevalence of plasma cells in lymphocytic infiltrates in untreated RA patients compared with non-RA patients with a different distribution [36]. Whether implant wear is inducing a different reaction in RA versus non-RA patients may potentially have implications for combination and immune targeted treatment of inflammation and osteolysis in these patients.

## 3. RANKL-RANK-OPG Axis

Receptor activator NF kappa B ligand- (RANKL-) RANK signalling has several important roles in the immune system and bone [13, 44–46]. Physiologically, RANKL is required for normal development of lymph nodes [13], as evident in knockout mice. In bone, RANKL interaction with its receptor, RANK, expressed by the osteoclast, induces the transcription factor nuclear factor of activated T cells-1 (NFATc1) [47–49]. NFATc1 is an essential factor for differentiation, multi-nucleation and activation [47–49]. NFATc1 binds directly to and regulates osteoclast differentiation genes such as tartrate resistant acid phosphatase (TRAP) [50], cathepsin K (Cath K) [51], osteoclast-associated receptor (OSCAR) [52], *β*3-integrin [53, 54], and calcitonin receptor (CTR) [50]. NFATc1 is also involved in autoregulation of itself, further enhancing gene expression and osteoclast differentiation [55].

RANK-RANKL interaction is inhibited by the decoy receptor OPG [12] and thus the ratio of RANKL to OPG has a crucial influence on bone resorption [56]. Interactions between RANK expressing cells of the lamina propria and T cells expressing RANKL also play a role in intestinal inflammation [57]. In the vasculature, RANKL interacts with RANK to promote survival of endothelial cells [58]. Additionally, RANKL is upregulated in the keratinocytes of inflamed skin [59]. Further to this, in an inflammatory arthritis model reminiscent of RA, activated T cells exacerbate joint destruction via RANKL upregulation [46].

### 3.1. RANKL-RANK-OPG in Bone Pathologies

The RANK-RANKL-OPG axis is known to regulate not only normal bone physiology but also alterations in RANK-RANKL-OPG interactions that play a role in bone disease. Uncoupling in the balance between the level and activity of these molecules culminates in osteoporotic or osteopetrotic phenotypes due to an increase or decrease in osteoclast formation and activity. This is particularly evident in focal bone loss associated with chronic inflammatory diseases such as rheumatoid arthritis, periodontal disease, and periprosthetic bone loss. In active RA, periodontal disease, and prosthetic loosening, elevated levels of RANKL relative to OPG are observed in the synovial-like soft tissue, gingival tissue, and soft tissues adjacent to sites of osteolysis [5–10]. Further to this the elevated ratio of RANKL : OPG expression is associated with increased differentiation and activity of the bone-resorbing osteoclasts [5, 7–9, 32, 60], suggesting RANKL : OPG ratio as an important indicator for bone erosion.

As RANKL and OPG are key molecules regulating bone loss in diseases, therapeutic interventions targeting these molecules and their signaling cascades are being investigated to treat a wide range of diseases.

### 3.2. RANKL-RANK-OPG in RA

Osteoclasts are the prominent cell eroding bone in inflammatory arthritis [20]. A seminal paper in the field of osteoimmunology used a RANKL knockout background to demonstrate that animals developed an osteopetrotic phenotype and a reduction in bone erosion, characterized by the absence of osteoclasts, whilst inflammation did not differ between wild-type and RANKL knockout mice [61]. In contrast, cartilage erosion was present in both control littermate and RANKL knockout mice [61], suggesting that the RANK-RANKL-OPG axis does not directly affect cartilage metabolism.

In human studies, the levels of soluble RANKL have been found to be higher than OPG in synovial fluids from patients with RA compared with osteoarthritis (OA) patients [62] suggesting a role in increased bone resorption. In support of this, a more recent large center study has reported the ratio of RANKL : OPG and markers of bone and cartilage degradation (such as collagen terminal 1 (CTX-1)) to be predictive of progression of radiological bone damage in RA [60].

In synovial tissues from patients with active RA, RANKL expression is predominantly located in sublining regions [62, 63] concentrated at focal sites of osteoclastic bone erosion in the pannus- bone interface [64]. In contrast, OPG has been described as being in regions of synovium some distance from the sites of bone erosion in RA [64]. We reported OPG is associated with endothelial cells and macrophages in the synovial lining layer of OA and normal tissues whilst absent in patients with active RA [63]. RANKL expressing cells have been detected in a subset of fibroblast-like synoviocytes and infiltrating mononuclear cells [62]. Further to this, activated synoviocytes from RA tissue express RANKL and have decreased OPG and are capable of supporting osteoclastogenesis* in vitro* [65]. RANKL expressing cells are also seen within areas of lymphocyte infiltration and dual immunostaining by ourselves, and others have shown that many of the RANKL-positive cells are a subset of CD3+ and CD4+ cells [6, 62, 63]. Activated T cells from RA patients have increased RANKL are able to induce osteoclast formation* in vitro* [62]. This study also reported a higher ratio of soluble RANKL relative to OPG suggesting T cells as a source of soluble RANKL in RA [62].

NFATc1 is a transcription factor crucial to RANKL-RANK signaling in the osteoclasts [47] and is initially identified as being expressed by T cells and involved in regulation of cytokine transcription [66]. We observed NFATc1 positive cells in lymphocyte aggregates in RA tissues [67]. Many of the NFATc1-immunostained mononuclear cells observed were single nucleated and thus could be either precursor cells of osteoclasts such as macrophages, or lymphocytes. Those with lymphocyte morphology are most likely activated T cells as most of them demonstrated NFATc1 positive staining localized mainly in nucleus [67]. These cells may promote osteoclastogenesis through the RANK/RANKL pathway [39] because as already mentioned, as activated T-cells demonstrate elevated expression of membrane-bound RANKL with the ability to support osteoclastogenesis* in vitro* [62].

### 3.3. RANKL-RANK-OPG in Periodontal Disease

The relative ratio of RANKL to OPG is also a significant indicator in bone loss associated with periodontal disease [5, 69]. Soluble RANKL is significantly higher in gingival crevicular fluid (GCF) of periodontitis patients than in healthy GCF, while OPG is not [69]. Similar to RA, B and T lymphocytes express RANKL in gingival tissues associated with periodontitis [5, 69] with expression of more than 50 and 90% of T cells and B cells, respectively. Consistent with a role in osteoclast regulation, lymphocytes isolated from gingival tissues of patients induced differentiation of mature osteoclast cells in a RANKL-dependent manner* in vitro* [69]. These results suggest that activated T and B cells can be the cellular source of RANKL and an inducer of bone resorption in periodontal disease.

In a crude mRNA analysis of tissue from dental patients, those with periodontitis exhibited significantly higher NFATc1 gene expression, compared with healthy subjects. Interestingly, NFATc1 and RANKL expression levels strongly correlated with each other and with clinical periodontal parameters [70].

### 3.4. RANK-RANKL-OPG in Peri-Prosthetic Osteolysis

Aseptic bone loss adjacent to orthopedic joint implants is a common cause of joint implant failure in humans. RANK, RANKL, and tumour necrosis factor (TNF-*α*) are key modulators of bone turnover and their expression has been reported by ourselves and others in the tissues near periprosthetic osteolysis in patients undergoing revision of total hip prostheses [9, 71, 72]. These factors were strongly expressed by large multinucleated cells containing polyethylene wear debris in revision tissues [9]. More interestingly a strong statistical correlation was found between volume of bone loss, polyethylene wear debris, and RANK, RANKL, and TNF-*α* expression [9]. This was consistent with the earlier findings of Stea et al. (2000) [72] where immunohistochemical detection of TNF-*α* positively correlated with radiographic scores of osteolysis.

In periprosthetic osteolysis, elevated levels of RANKL, relative to its competitor OPG, are associated with increased differentiation and activity of the bone-resorbing osteoclasts [8, 32, 73]. An earlier study had shown that cells isolated from periprosthetic tissues containing wear particles expressed mRNA encoding for the proosteoclastogenic molecules, RANKL, its receptor RANK, monocyte colony-stimulating factor (M-CSF), interleukin- (IL-) 1 beta, TNF-*α*, IL-6, and soluble IL-6 receptor, as well as OPG [8]. Other studies showed that osteoclasts formed from cells isolated from periprosthetic tissues in the presence and absence of human osteoblastic cells* in vitro* [8, 74]. When osteoclasts formed in the absence of osteoblastic cells, markedly higher levels of RANKL mRNA relative to OPG mRNA were expressed. Particles of prosthetic materials also stimulated human monocytes to express both osteoclast-associated genes and osteoclast mediating factors* in vitro* [8]. These findings suggest that ingestion of prosthetic wear particles by macrophages results in expression of osteoclast-differentiating molecules and stimulation of macrophage differentiation into osteoclasts [8]. Subsequent immunohistochemical studies demonstrate significantly higher levels of RANKL in the periprosthetic tissues of patients with implant failure than in similar tissues from osteoarthritic and healthy subjects [32]. In contrast, OPG protein levels were similar in all tissues with the net result of higher RANKL : OPG ratio [32]. Of interest, RANKL protein and mRNA were predominantly associated with macrophage cells containing wear particles in the periprosthetic tissues [32]. These findings support the contention that high levels of RANKL in periprosthetic tissues of patients with prosthetic loosening may significantly contribute to aseptic implant loosening [32].

We observed both mRNA and protein expressions of NFATc1 to be higher in periprosthetic osteolysis than in OA tissues although levels did not reach significance [75]. This is consistent with low T lymphocyte numbers observed in these tissues. This may provide an explanation why lower than expected NFATc1 protein and mRNA levels are found in periprosthetic osteolytic tissues.

## 4. The ITAM Pathway in Osteoimmunology

**Figure 1:**
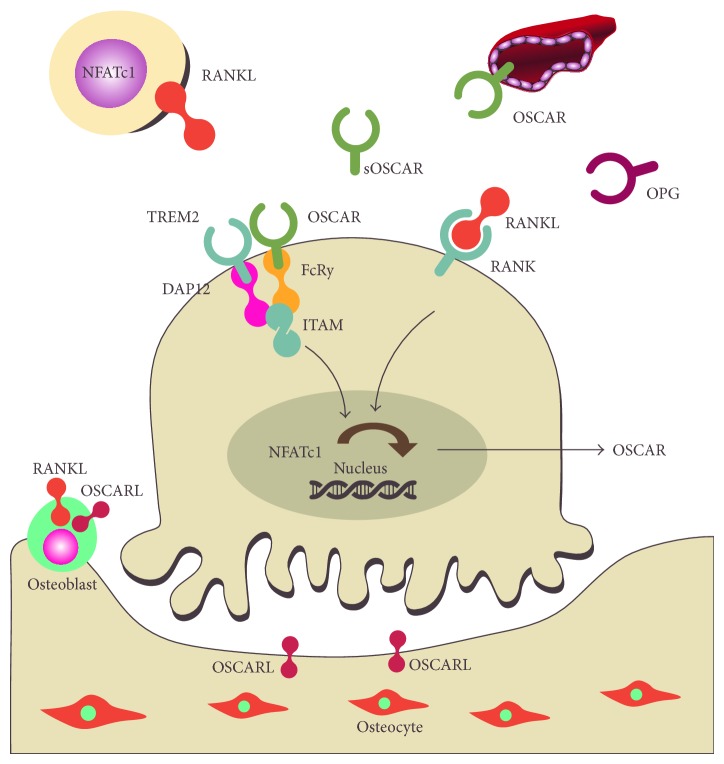
RANGL-RANK-OPG axis and ITAM, the costimulatory pathway, in inflammation induced localised bone loss pathologies.

The ITAM pathway regulates proliferation, survival, and differentiation of effector immune cells and provides osteoclasts with costimulatory signals [14, 76–80]. In preosteoclasts and osteoclasts, innate immune receptors, TREM2 and OSCAR, associate with the ITAM adaptor proteins DAP12 and Fc receptor gamma-chain (FcR*γ*), respectively [80, 81]. DAP12 and TREM2 are required for differentiation into multinucleated, bone-resorbing osteoclasts* in vitro* via phosphorylation of the Syk tyrosine kinase [79]. OSCAR on the cell surface mediates signal transduction via FcR*γ* [82, 83] (Figure 1). The induction of intracellular calcium via this pathway is required in conjunction with RANKL-RANK interaction for NFATc1 induction [84].

### 4.1. Mutations of ITAM-Associated Molecules and Bone Phenotypes* In Vivo*

In the context of human pathology the roles of TREM2 or DAP12 have only begun to be recognized. Studies in diseased tissues, particularly in Nasu-Hakola disease [85–87] and very recently in Alzheimer's disease [88], have shown that these molecules may be involved. Mutations in TREM2 or DAP12 have been associated with bone pathologies such as bone cysts and increased fractures (in addition to presenile dementia) in Nasu-Hakola disease [87, 89]. These studies support a role for DAP12 and a relationship between the skeletal and psychotic characteristics observed in Nasu-Hakola disease and for schizophrenia and presenile dementia [90]. In TREM2 deficient individuals the osteoclast precursors failed to differentiate into effective bone-resorbing cells [91]. Consistent with this, Paloneva et al. [87] demonstrated that function mutations in DAP12 and TREM2 result in an inefficient and delayed differentiation of osteoclasts* in vitro*. In postmenopausal osteoporosis a rare allele (G allele) of OSCAR-2322A&gt; G (SNP in the 5′ flanking region) has been associated with lower bone mineral density [92].

Although animal models are not the focus of this review it is interesting to note the phenotypes of ITAM related molecules in single and combination knockouts. TREM^−/−^ mice have an osteopenic phenotype similar to Nasu-Hakola disease.* In vitro*, lack of TREM2 impairs proliferation osteoclast precursors and affects the rate of osteoclastogenesis by accelerating differentiation into mature osteoclasts [93] suggesting different effects of knocking out TREM2* in vivo* and* in vitro*. In DAP12 deficient mice (−/−) there are an increased bone mass (osteopetrosis) and impeded development of osteoclasts. Mice that are double knockout for the adaptors DAP12^−/−^ and FcR*γ* ^−/−^ are severely osteopetrotic and bone marrow derived osteoclast precursors from these mice are unable to differentiate into mature osteoclasts in a RANKL- and M-CSF-mediated culture system [79, 82]. Although there is some redundancy, findings from these studies suggest that DAP12 is the predominant factor responsible for optimal osteoclast differentiation.

### 4.2. OSCAR Signalling and Function in Bone Regulation

OSCAR is an IgG-like receptor expressed by monocytes, macrophages monocyte-derived dendritic cells in humans, and is involved in antigen presentation as well as survival, maturation, and activation of dendritic cells [14, 76, 77, 83, 94, 95]. Ligation of human OSCAR on monocytes and neutrophils results in the induction of a proinflammatory cascade and the initiation of downstream immune responses [95]. Importantly, cell bound OSCAR on osteoclast precursors is an essential costimulatory factor in osteoclast formation but does not bypass the requirement of RANKL. RANKL-RANK induction of NFATc1 expression precedes that of OSCAR [96] and is crucial for induction of OSCAR gene expression [52]. In addition, ligand-activated OSCAR interacts with FcR*γ* to produce an increase in intracellular calcium [95] that augments NFATc1 expression [96]. This establishes a positive feedback loop that results in marked elevation of both OSCAR and NFATc1 expressions in terminal stages of osteoclast formation [52, 96]. These findings demonstrate a significant role for OSCAR in immune modulation as well as osteoclastogenesis.

*In vitro* studies demonstrate that addition of OSCAR-Fc to osteoblast-osteoclast cocultures results in the inhibition of osteoclast differentiation with Kim et al. (2002) suggesting that this was due to OSCAR-Fc blocking an osteoblast derived OSCAR ligand binding to OSCAR [14]. This may be in addition to the recent identification of the motifs within fibrillar collagens in the extracellular matrix (ECM) as OSCAR ligands [97]. The importance of OSCAR is further highlighted by the fact that soluble OSCAR (s)OSCAR, in the form of OSCAR-Fc, has also been shown to inhibit osteoclast differentiation from PBMCs in the presence of RANKL, M-CSF, and TGF-*β* [94].

Costimulatory immune pathways may further increase osteoclast differentiation and activity [81, 82], particularly in chronic inflammatory diseases with an immune component such as in RA, periprosthetic osteolysis, and periodontal disease. In fact, our studies [67, 75] and those of others suggest that deregulation of ITAM-associated molecules contributes to the pathogenesis and severity of rheumatoid arthritis, periodontal disease, periprosthetic osteolysis, and osteoporosis [10, 67, 75, 92, 94, 98, 99].

### 4.3. Expression of ITAM-Associated Molecules in Chronic Inflammation Induced Localised Osteolysis

We, and others, have demonstrated increased levels of ITAM-related factors, including TREM2, DAP12, OSCAR, and FcR*γ* in human periprosthetic tissues adjacent to sites of osteolysis [75] and in RA synovial tissues [67, 94]. Additionally, we have observed ITAM-related factors expressed in periodontitis tissue adjacent to bone loss (unpublished observations). Of these factors, soluble and membrane-bound OSCAR have been more extensively assessed in the context of RA and vascular disease (expanded on below).

### 4.4. Expression of ITAM-Associated Molecules in Rheumatoid Arthritis

We observed markedly higher levels of TREM2, DAP12, OSCAR, and FcR*γ* in active RA patients compared to synovial tissues from inactive RA, OA, or control healthy joint. Multiple cell types expressed TREM2 including mononuclear cells in lymphoid aggregates and fibroblasts [67]. In OA tissues, TREM2 immunostaining was noticed in monocyte/macrophage-like cells mainly around perivascular areas and on blood vessels (unpublished observations). The positive TREM2 immunostaining on the vasculature was consistent with the finding on expression of TREM2 in endothelial cells that has been documented earlier [100]. TREM2 immunostaining was also occasionally spotted on lymphocyte-like cells in some OA tissues; however, to date there has been no study indicating the expression of TREM2 in lymphocytes but further investigation is needed for confirmation.

Interestingly, DAP12 appeared predominantly associated with macrophage-like cells in the sublining of the synovial tissue, particularly in the macrophage-like cells in the lining of the OA group [67]. More recently, a study by Chen et al. (2014) [101] reported that mRNA expression levels of DAP12 in the peripheral blood mononuclear cells of active RA patients were significantly higher in active RA patients than in inactive RA or OA patients. This is consistent with our observations [67] of higher levels of DAP12 protein expressed in the synovium in active RA patients than in inactive RA or OA patients. They also noted that the levels significantly decreased after effective therapy [101].

FcR*γ* protein associates with fibroblasts and monocytes of the synovial sublining whilst lymphoid aggregates and the vasculature do not express FcR*γ* [67]. Of note, similar to DAP12, FcR*γ* was associated with macrophage-like synoviocytes in the synovial lining with some scattered monocytes in the sublining of the OA tissue. This increased DAP12 and FcR*γ* expression might indicate a role in the pathogenesis of OA but this is yet to be determined [67].

### 4.5. Soluble and Synovial Tissue Levels of OSCAR in RA

**Figure 2:**
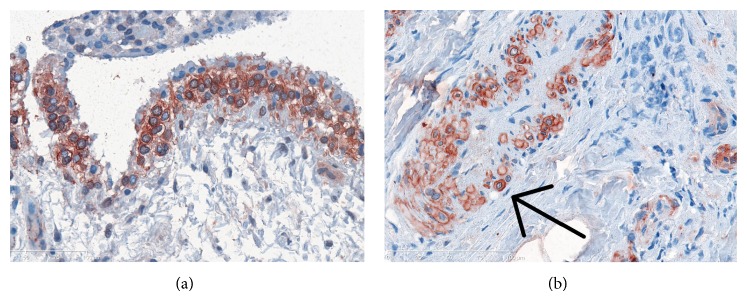
OSCAR positive cells (red) in synovial tissue. (a) OSCAR immunostaining in the lining cells of OA tissues. (b) Mononuclear OSCAR positive cells as indicated by arrow. The magnification was 400x.

Analysis of human synovial tissue, serum, plasma, and synovial fluid suggests that OSCAR expression is associated with disease activity in RA [67, 94, 98, 99]. Recent studies show that OSCAR protein expression is increased in monocytes from RA patients compared with healthy individuals, correlating with inflammatory disease activity (DAS28) [94]. OSCAR has also been noted to be expressed by mononuclear cells adjacent to synovial microvessels in RA tissues [94]. Consistent with these findings, our immunohistochemical studies show that high levels of OSCAR are associated with mono- and multinuclear cells in active RA tissues compared to tissues from OA and normal patients [67] (Figure 2). Furthermore, semiquantitative analysis confirmed that there is a significant elevation of OSCAR (*P* < 0.05) in active RA synovial tissues compared to osteoarthritis synovial tissues. This increased expression of OSCAR in the synovial tissue of active RA suggests OSCAR regulation by inflammatory cytokines and supports a role for OSCAR in the pathogenesis of RA.

A study investigating the clinical, radiological, and synovial immunopathological responses following antirheumatic treatment in RA proposed that high synovial tissue vascularity predicted favorable clinical and radiological responses to treatment [102]. Similar to this, we previously reported the increased OPG staining associated with the vasculature in synovial tissues retrieved from the patient in remission, OA and normal compared with active RA [63]. Furthermore, we have reported increased levels of OPG associated with vasculature following treatment with DMARDS [7].

More recently we have detected increased expression of OSCAR protein associated with the microvasculature of synovial tissue from all inactive and active RA patient tissues (9/9) compared to none in the normal synovial tissue group (0/9) [67]. Importantly, in diseased tissues OSCAR was expressed mostly on the luminal side of the microvasculature, consistent with OSCAR expression by endothelial cells [67, 103]. Our findings suggest that OSCAR is associated with the endothelial cells of the microvasculature and is either produced by endothelial cells or secreted by other cells and bound by the endothelial cells in inflammatory states. The marked reduction in the OSCAR associated with endothelial cells observed in OA and healthy synovial tissues compared with active RA indicates an immune modulatory mechanism [67], which may also signal back to the osteoclasts and regulate bone resorption.

Following observations of OSCAR association with blood vessels the expression of OSCAR was investigated in endothelial cell lines* in vitro*. Our analysis on bone marrow-derived endothelial cells (BMECs) challenged with IL-1*β* and TNF-*α in vitro* demonstrated that the inflammatory cytokines increased OSCAR expressed as both mRNA and secreted and membrane-bound proteins [67]. Together with* in vivo* observations on synovial tissues and serum levels these studies suggest that inflammatory cytokines in RA regulate cleavage or secretion of sOSCAR from preosteoclasts or the microvasculature.

The regulation by inflammatory cytokines, such as TNF-*α*, of OSCAR messenger RNA expression has also been observed in monocytes [94]. Interestingly, levels of OSCAR were found to increase in serum from RA patients following anti-TNF treatment [94]. Of note, these studies did not investigate gene or the release of protein OSCAR by human peripheral blood derived osteoclasts in response to TNF-*α* in conjunction with RANKL.

### 4.6. Expression of ITAM-Associated Molecules in Periodontal Disease

To our knowledge, very limited descriptive or functional studies have investigated ITAM factors in periodontitis and normal gingival tissues. An early study however reported that isolated polymorphonuclear neutrophils from GCF of adult periodontitis patients exhibited higher Fc alpha RI and Fc gamma RI levels and lower Fc gamma RIIa and Fc gamma RIIIb levels than peripheral blood polymorphonuclear neutrophils. They found that GCF derived polymorphonuclear neutrophils had a reduced ability to phagocytose and kill IgG1-opsonized* P. gingivalis* compared to peripheral blood polymorphonuclear neutrophils [104].

**Figure 3:**
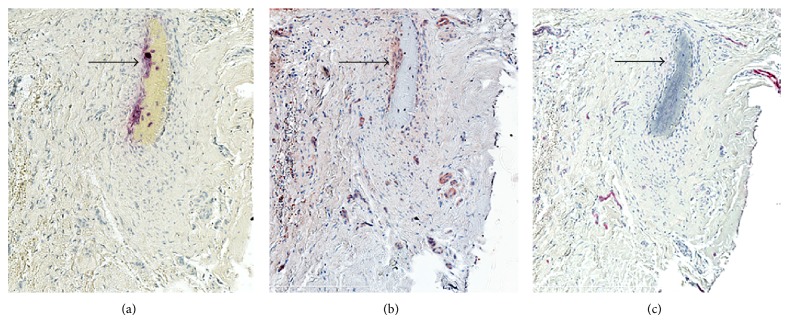
Expression of osteoclast and vascular-associated molecules in mildly inflamed gingival tissues. (a) TRAP (osteoclast marker), (b) OSCAR C. Von Willebrand factor to identify the microvasculature. The magnification was 200x.

Our recent unpublished observations have identified OSCAR colocalizing with TRAP in cells in serial sections of mildly inflamed gingival tissue (Figure 3). Of note, these osteoclast markers are highly expressed in the multinucleated cells on the bone. Similar to the previous observations of expression of ITAM-associated molecules in active and inactive RA patients OSCAR expression was also noted in the microvasculature. Given the similarities in pathogenesis of RA and periodontitis [27] it is worth investigating expression of ITAM factors in periodontitis, gingivitis, and normal gingival tissues.

### 4.7. Expression of ITAM-Associated Molecules in Peri-Prosthetic Osteolysis

**Figure 4:**
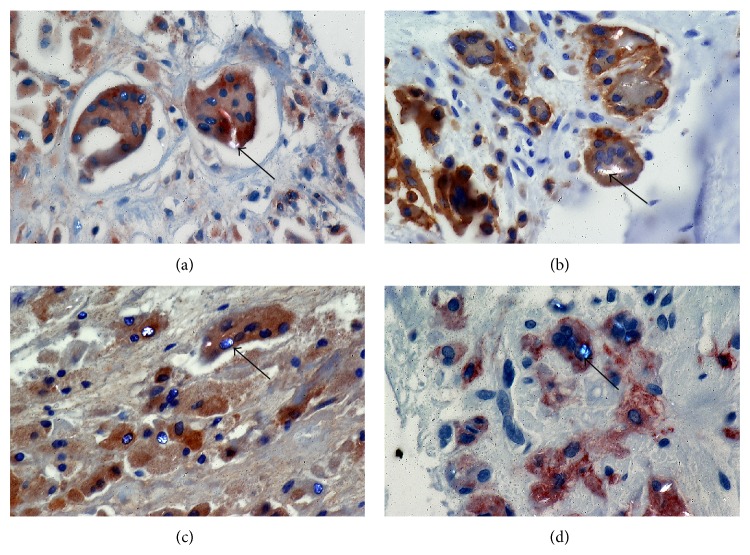
Expression of ITAM-associated molecules in PE-containing tissues from sites of aseptic loosening due to osteolysis. (a) TREM2, (b) DAP12, (c) OSCAR, and (d) FcR*γ* immunostaining. The magnification was 400x.

We have reported a marked increase in the levels of TREM2 and DAP12, OSCAR, and FcR*γ* in tissues containing PE particles, compared with OA synovial control tissue when assessed by a semiquantitative scoring system [75]. Furthermore, the observed increased levels of these proteins in peri-prosthetic tissues were consistent with the finding that the corresponding mRNA levels were also increased [75]. Of interest, PE-containing osteoclast-like cells in these tissues were associated with high levels of TREM2 and OSCAR protein and their respective adaptor molecules DAP12 and FcR*γ* [75] (Figure 4). Consistent with these* in vivo* observations, PE particles added to human peripheral blood derived osteoclast cells* in vitro* upregulated ITAM expression [75].

It is important to also understand the role synovial fluid may play in modulating regulators of cartilage and bone destruction in the joint. Andersson et al. (2007) found that synovial fluid from patients with OA stimulated the mRNA expression of OSCAR and NFATc1 in mouse calvarial implants* in vitro*, while mRNA expressions of DAP12 and FcR*γ* were not affected by synovial fluid from either revision or OA patient groups. The authors suggested that perhaps OSCAR and NFATc1 mRNA might be regulated by soluble factors that are present in OA synovial fluid. However, expression of DAP12 and FcR*γ* was not regulated in the same way [105]. This is an area that could further be explored.

Considering that inflammation recruits osteoclast precursors and can induce the differentiation and activation of osteoclasts the enhanced expression of ITAM-related molecules in revision tissues could exacerbate bone loss in this disease.

### 4.8. Potential Role for OSCAR in the Clinic

Previous studies have demonstrated that soluble fusion (OSCAR-fc) protein, comprising the extracellular domain of OSCAR, could inhibit osteoclastogenesis in murine preosteoclast/osteoblast cocultures [14] and PBMCs cultured in the presence of RANKL, M-CSF, and TGF-*β* [94]. In this situation sOSCAR could compete with OSCAR ligand and reduce OSCAR signalling. The ability of soluble sOSCAR* in vitro* to impede osteoclast formation may prove useful in inhibiting osteoclast differentiation and may thus prevent bone damage in diseases such as RA.

There is conflicting data as to whether sOSCAR increases in healthy individuals or it increases as a result of erosive activity in RA. Soluble OSCAR has been detected in serum and reported to be higher in healthy compared to RA patients [94, 99]. Serum levels of sOSCAR were shown to inversely correlate with erosion and disease activity [99]. A recent study, however, reported higher levels of sOSCAR in the plasma of RA patients rather than healthy individuals [98]. We have also detected sOSCAR in the synovial fluid of OA and active RA patients with no significant difference between these diseases [67]. We believe that sOSCAR has the potential to act as decoy receptor for OSCAR ligand within the joint and affect osteoclast development in RA. It is possible that successful treatment results in increased cleavage of cell associated OSCAR resulting in increased sOSCAR levels in the joint. In this way sOSCAR regulates osteoclastic bone resorption and is an early marker that predicts joint damage. The biological effect of serum and synovial fluid-derived OSCAR on osteoclastogenesis is yet to be investigated.

Synovitis and erosion are not always linked with some patients having progressive erosive disease despite being in remission and it is unclear what factors drive this [106]. The discordance between clinical inflammatory disease activity and radiological outcomes emphasizes the need for a validated marker of bone damage in conjunction with current clinical parameters that are routinely assessed [107, 108]. The ability to monitor bone erosion will allow the clinician to make important decisions on therapy earlier [109] and reduce structural joint damage [110]. Currently unaddressed RA-induced joint damage affects mobility of patients later in life and predisposes to secondary osteoarthritis [110]. In addition, while we have numerous markers that reflect inflammation and related disease activity there are none that monitor joint erosion, other than X-rays which only indicate damage after it has occurred [111]. Therefore, it is essential that an accurate early marker of joint erosion is identified in order to guide effective treatment modalities in order to protect the joint of arthritic patients.

## 5. Conclusion

While the significance of ITAM-associated molecules has been largely established in the context of bone biology and an immunological point of view, limited studies have been carried out on osteoclast ITAM-related molecules in human bone pathologies. The increased levels of ITAM factors in inflamed tissues adjacent to sites of localized bone loss in RA, periodontal disease, and periprosthetic osteolysis may prove indicative of the disease progression. Further to this, levels of the soluble factor, OSCAR, in serum or local fluid, may provide us with a potential bone destructive marker and potential target for modulation of bone erosion.
